# Which Patients with Chronic Periprosthetic Joint Infection Are Less Suitable to Successful Two Stage Exchange Arthroplasty Surgery? A Retrospective Clinical Trial

**DOI:** 10.3390/clinpract13010017

**Published:** 2023-01-28

**Authors:** Alberto Di Martino, Gabriele Di Carlo, Davide Pederiva, Valentino Rossomando, Federico Pilla, Matteo Brunello, Claudio D’Agostino, Leonardo Tassinari, Eleonora Zamparini, Cesare Faldini

**Affiliations:** 1 Ist Orthopaedic Department, IRCCS—Istituto Ortopedico Rizzoli, via Giulio Cesare Pupilli 1, 40136 Bologna, Italy; 2 Department of Biomedical and Neuromotor Science-DIBINEM, University of Bologna, 40123 Bologna, Italy; 3 Infectious Diseases Unit, IRCCS Azienda Ospedaliero Universitaria di Bologna—Policlinico di Sant’Orsola, 40138 Bologna, Italy

**Keywords:** chronic periprosthetic hip infection, two-stage exchange failure, depression, polymicrobial PJI

## Abstract

Background: Two-stage exchange (TSE) arthroplasty is currently considered the gold standard for chronic periprosthetic joint infections (PJIs), despite a failure rate reported in up to 10% of patients. Little is known about the risk factors that may compromise successful TSE arthroplasty management in such patients. The main purpose of the current study was to highlight the potential risk factors of patients with chronic PJIs after THA managed by implant removal, outlining the differences between reimplanted patients and those that were never reimplanted because of a non-eradicated infection. Methods: We conducted a retrospective observational study of patient candidates for TSE arthroplasty surgery, managed at the authors’ institution, over a four-year timeframe. The data were retrieved from the hospital’s information database. The enrolled population was divided into two Groups: A, reimplanted; B, non-reimplanted because of a non-eradicated infection within one year. For each Group, demographic information, PJI-related risk factors, type of pathogen and presence of single or polymicrobial infection, were collected and analyzed. Results: In total, 21 patients were included in the study, 14 patients in Group A and 7 in Group B. Major Depression (*p* = 0.049) and polymicrobial infection (*p* = 0.04) were more commonly observed in patients that were not reimplanted in the study period. No differences between the two groups were observed when other characteristics were compared. Conclusions: Patients with major depression, or those hosting polymicrobial periprosthetic hip infections, are more susceptible to failure of TSE arthroplasty procedures for chronic PJIs, hampering THA reimplantation. Current findings may drive further research and contribute to the understanding of the role of these risk factors in chronic PJI patients.

## 1. Introduction

Periprosthetic joint infections (PJIs) remain one of the most feared complications in total hip arthroplasty (THA) [1], with an incidence ranging from 0.5 to 3%. More than one million THAs are performed yearly [2] with an increased 400% volume of this procedure expected by 2030 [3]. According to these figures, an increase in the number of PJIs is expected in the future [4,5].

While there is no commonly agreed diagnosis of PJI [6], diagnostic criteria were developed in 2018 to overcome the shortcomings of previous classifications that represented a consensus, rather than an evidence-based, methodology [7,8,9]. Chronic PJIs are diagnosed at least four weeks after THA surgery [10]. This temporal criterion is related to the formation of biofilm, which is a consortium of microorganisms embedded in a matrix that adheres to the prosthesis, creating a microenvironment that protects the pathogens from antibiotic treatment and from the patient’s immune system [11]. Therefore, patients with chronic infections after THA require aggressive treatments that include the removal of the implant and prolonged antibiotic therapy. Two-stage exchange (TSE) arthroplasty is currently regarded as the gold standard in the treatment of chronic PJIs [12,13,14,15]. It is based on two separate surgeries: the first is a resection arthroplasty, in which the implant is removed and an extensive debridement is performed. After surgery, systemic antimicrobials are administered, according to the sensitivity profile of the isolated microorganism, or empirically when no pathogen is identified [13,16,17]. Reimplantation occurs if complete eradication of the infection is achieved [10].

The TSE procedure is successful in over 90% [10,18] of patients with chronic PJI; however, according to these data, a percentage of patients remains chronically infected and is never reimplanted. The understanding of the role of specific risk factors associated with a worse outcome and the failure of TSE procedures could be useful for physicians managing chronic PJIs. At present, a BMI > 30 kg/m^2^, ASA > 2, diabetes mellitus, alcohol abuse, smoking habits, active infection [19], rheumatoid arthritis, malignancy [20], intra vascular drug abuse (IVDA) [21], revision surgery [22], cardiovascular disease (CVD), and depression [20] are known risk factors for the occurrence of PJI after THA implant. However, little is known about the effect of these variables on the success of TSE arthroplasty surgery.

The purpose of the current study was to investigate demographics, potential risk factors and microbiological characteristics of patients with chronic PJIs as candidates for TSE arthroplasty of the hip.

## 2. Materials and Methods

The present study was designed as a retrospective observational study of patient candidates to TSE arthroplasty surgery, performed at the authors’ institution, from 1 January 2016, to 31 December 2019. The study included patients who were >19 years of age, in which TSE was considered to be the treatment of choice. The exclusion criteria were the following: patients treated surgically at another hospital, and patients without complete documentation. TSE arthroplasty surgery was considered successful if the patient was reimplanted within 1 year from resection arthroplasty surgery. Ethical committee approval (CE AVEC: REVIAN/229/2021/Oss/IOR) was obtained before patients’ enrolment to the study.

Patients’ recruitment was performed by requesting from the hospital information database the retrieval of all the procedures performed in the 4-year timeframe. A total of 21 patients with a diagnosis of chronic PJI on THA were included in the study. Periprosthetic infections were initially diagnosed by evocative clinical signs, such as pain at the site of the prosthesis. Patients were then analyzed by imaging where signs of prosthetic loosening could be observed. Serum markers, such as Erythrocyte sedimentation rate (ESR) and C-reactive protein (CRP) levels, while nonspecific, were measured. Intraoperative extemporaneous histological examination was then performed. Finally, intraoperative histological and culture examinations were performed, that were considered positive if more than five neutrophils per high-power field were present in five high-power fields observed by histological analysis of the periprosthetic tissue at ×400 magnification The latter was considered positive with at least 2 positive cultures of the same organism.

**Figure 1:**
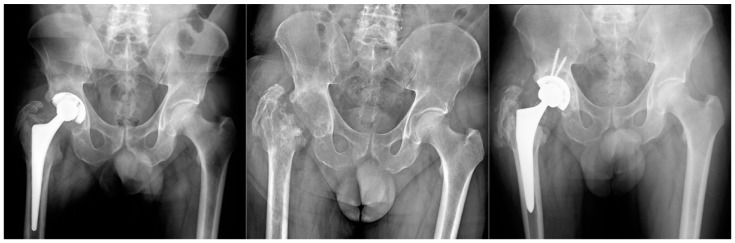
Group A Patient affected by chronic PJI treated with TSE. The patient was eligible for reimplantation after 4 month targeted antimicrobial therapy. After 12 months follow up there were no signs of recurrence of infection.

**Figure 2:**
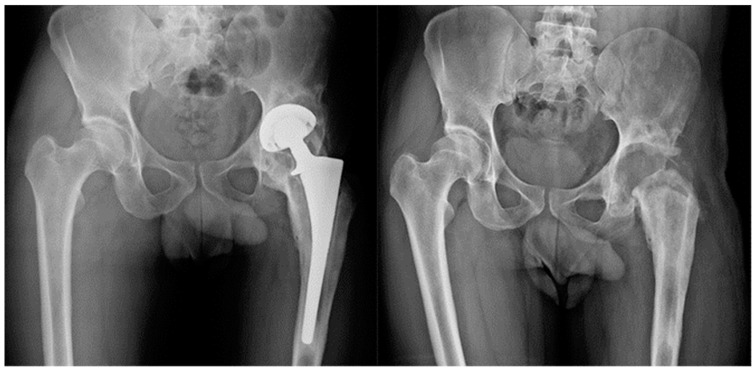
Group B. Patient with communicating sinus tract at the diagnosis. After ex-plantation and targeted antimicrobial therapy for a polymicrobial PJI, the patient was not considered eligible for reimplantation.

The same procedure was performed in all patients. Patients were explanted and antibiotic therapy was subsequently set up, at first empiric, and then targeted for the isolated pathogen. In no case were antibiotic spacers used. The antibiotic therapy was administered according to the culture sensitivity results. Antibiotic therapy was performed until normalization of CRP following explantation and, in any case, never for more than 8 consecutive weeks. One year after resection arthroplasty was performed, the following two possible outcomes were evaluated: successful TSE arthroplasty was performed in 14 patients (Group A) (Figure 1), and resection arthroplasty without reimplantation in 7 (Group B) (Figure 2). The following were the replanting criteria: CRP normalization with antimicrobial treatment in at least two controls separated by two weeks. If normalized, antibiotic therapy was discontinued, patients were then monitored for an additional 4 weeks by weekly CRP checks. If the latter remained normal, labeled leukocyte scintigraphy was done. If the latter was also negative, reimplantation was scheduled, which was to be performed only after intraoperative negative histology.

A retrospective analysis was performed on these patients, outlining the differences between reimplanted patients and those that were never reimplanted because of treatment failure, defined as the persistence of, or recurrence of, infection with the initial causative bacteria or infection with a new bacterium, with or without the presence of the initial causative bacteria at any time during follow-up [23]. Antibiotic therapy was performed until CRP normalization following explantation and, in any case, never for more than 8 consecutive weeks. All surgeries were performed by the hip surgery team consisting of 4 senior surgeons. All patients underwent physiotherapy treatment following explantation: in particular, patients were encouraged to gain the ability to walk independently, at first with an ambulator, assisted by a physical therapist, and then with the use of crutches. Patients in group A then underwent treatment again to perform reimplantation, in contrast to patients in group B who were not reimplanted.

Clinical records were reviewed to assess general patients’ demographics, presence of risk factors associated to the occurrence of PJI and data about pathogens that sustained the infection.

Descriptive statistics were used to report demographics (gender, age at the primary THA, primary diagnosis supporting THA performance, surgical approach, irrigation and debridement) and patient-related risk factors potentially associated to an increased risk of PJI (BMI > 30 kg/m^2^, ASA > 2, diabetes mellitus, alcohol abuse, smoking habits, active infection at other sites, rheumatoid arthritis, malignancy, intra vascular drug abuse (IVDA), revision surgery, cardiovascular disease (CVD), major depression) [19,20,21,22,24,25] when available.

Data about pathogens that sustained the infection (isolation, positive or negative gram stain, and presence of polymicrobial infection) were collected for each subgroup. Differences between Group A and Group B were analyzed by means of a Chi-square test, performed to study the distribution of selected variables. Sensitivity was set at *p* < 0.05.

## 3. Results

### 3.1. Demographics and Population Characteristics

Group A accounted for 10 males and 4 females with an average age at the time of THA surgery of 51 years (range 31–82 years). The patients were treated after the diagnosis within an average time of 3 months (range 2 months–6 years). Six patients underwent THA for degenerative arthritis, 4 for an unknown cause, 2 after trauma, 1 for avascular necrosis of the hip (AVNH) and 1 for secondary arthritis in developmental dysplasia of the hip (DDH). The surgical approach at first surgery was posterior–lateral (PL) in eleven patients, and direct lateral (DL) in three. For patients in Group A the mean duration from resection arthroplasty to reimplantation was 6.7 months (range 3–12 months). In 8 out 14 patients one irrigation and debridement (I&D) procedure was performed after an average of 6.5 months (range 3–10) from explant.

Group B accounted for 4 male and 3 female patients with an average age of 57.7 years (range 27–86). In 3 out of 7 patients the indication for THA was unknown, 2 underwent THA after trauma, 1 for degenerative arthritis and one for DDH. THA was performed in 4 patients by means of the PL approach, in 2 patients by a DL approach, and in 1 patient by a Direct Anterior (DA) approach. Patients in Group B underwent THA explantation and targeted antimicrobial therapy. In 5 out 7 patients I&D was performed after 3.4 months from explant, and 4 of these had another I&D at 10 months from the first procedure. Group B patients were not reimplanted during the study timeframe (Table 1).

### 3.2. Risk Factors

Risk factors were retrieved by the clinical records. In Group A the relevant facts were the following:, the average BMI at the time of explant surgery was 26 (range 23–29); 6 patients had an ASA Score > 2; 4 patients had Diabetes Mellitus; 5 patients were smokers; 3 patients had an infection at other sites when the PJI was diagnosed (2 had urinary tract infections and 1 had a septic shock); 1 had a positive oncological history for breast cancer; 2 patients had positive intra-venous drug abuse history; 7 out of 14 underwent at least one revision surgery before the diagnosis of PJI; 4 had a cardio-vascular disease, being hearth failure (HF) in 3 patients, and atrial fibrillation (AF) in 1; 1 patient had mood disorders (major depression).

In Group B the relevant facts were the following:, the average BMI at surgery was 25 (range 23–28); 3 patients had an ASA Score > 2; there was 1 patient with Diabetes Mellitus; 3 patients were smokers; 2 had an active infection at another site (bronchopneumonia and urinary tract infection, respectively); 1 had a positive oncological history (Chronic Lymphoproliferative syndrome); 1 patient had a positive history of intra venous drug abuse; 2 patients had one revision surgery before being referred to the authors’ institution; 1 patient had cardio vascular disease (heart failure); 3 had mood disorders (major depression) (Table 2).

Patients in Group B were significantly more affected by major depression (*p* = 0.049) compared to patients in Group A. There was no significant difference between groups in terms of obesity (BMI > 30; *p* = 0.59), ASA Score > 2 (*p* = 1), Diabetes Mellitus (*p* = 0.46), smoking habits (*p* = 0.75), presence of known infectious diseases at distant organs (*p* = 0.71), history of malignancy (*p* = 0.59), intra-venous drug abuse (IVDA) (*p* = 1), history of revision surgery (*p* = 0.34), and presence of associated cardiovascular disorders (CVD) (*p* = 0.46).

In both groups, no patients were alcohol abusers or suffered from rheumatic diseases.

### 3.3. Pathogens

Group A had 10 infections sustained by gram+ germs, 2 sustained by gram−, and 2 polymicrobial infections. Group B contained 2 PJIs sustained by gram+ pathogens, 1 by gram− germs, and 4 were polymicrobial. Patients in Group B were significantly more affected by polymicrobial infections, compared to patients in Group A (*p* = 0.04) (Table 3).

#### 3.3.1. Gram+ Subgroup

In Group A, in 10 patients with PJI supported by gram+ germs, the infection broke out, on average, 50.9 months after primary THA surgery. Of the patients, 4 required I&D after 3.6 months from explant surgery. On average, in this subgroup, re-implantation occurred after 6.4 months. In all, 6 patients did not require I&D and were re-implanted at an average of 4.2 months.

In 2 Group B patients PJI was sustained by gram+ germs, and the infection broke out, on average, 73.5 months after primary THA surgery.

#### 3.3.2. Gram− Subgroup

In Group A, 2 patients had PJI supported by gram− pathogens. The infection broke out, on average, after 72 months from the first surgery. They needed I&D, on average, 3 months after explant; reimplantation occurred, on average, after 8.5 months.

One patient in Group B had PJI sustained by gram− pathogen, and the infection was diagnosed two months after surgery.

#### 3.3.3. Polymicrobial Subgroup

In Group A, 2 patients had polymicrobial PJI, and the infection broke out after 6 weeks from THA. Treatment consisted, for both patients, of TSE and targeted antibiotic therapy with I&D after 10 months. Reimplantation was performed after 11 months.

In Group B, 4 patients had PJI sustained by a polymicrobial infection, which occurred an average of 84.5 months after the first surgery.

## 4. Discussion

The present study compared demographics, prevalence of risk factors and germ characteristics between two groups of patients suffering from chronic PJI on THA managed by TSE arthroplasty. The aim of the study was to highlight possible risk factors for the failure of the procedure, which meant that the patient was not reimplanted 1 year after resection arthroplasty. Patients with successful TSE arthroplasty (Group A, *n* = 14) showed no recurrence of PJI at 1 year follow up. This finding was consistent with the success rate of two-stage exchange strategy available in the literature, which was reported to be above 90% [10,26,27]. Seven patients (Group B) were managed by removal of the implant and targeted antimicrobial therapy, but they were not considered eligible for reimplantation; patients in this group were significantly more affected by polymicrobial infections and major depression.

This study had some limitations; first, it was a retrospective study, and there are inherent limitations to this design. However, it offers interesting clues to guide future prospective works. Second, due to the low incidence of PJI in the THA population, the size of the study population was relatively small.

Demographic analysis showed that most patients in this study were males (71% Group A and 57% Group B), with a mean age of 51 years in Group A and 57.7 years in Group B. It is still under debate if gender could be considered a risk factor for PJI on THA. Chen et al. [28] did not demonstrate any association between gender and risk of infection. A meta-analysis conducted by Kong et al. [19], that included 24 studies investigating patient-related, surgery-related and comorbid conditions, found that male gender was a risk factor for PJI on knee arthroplasty. Regarding the impact on TSE outcomes, Cancienne et al. [29] reported that female gender was associated with a higher risk of not being reimplanted after resection arthroplasty.

There is no agreement on the fact that age of the patients at surgery could be a risk factor for PJI. Resende et al. [30], performed a systematic review analyzing several potential risk factors for PJI. The study included 37 manuscripts about 22,689 PJI patients, and outlined that older age was a protective factor for PJI. This result was in contrast with the findings of Kunustor et al. [31], who did not report any association between age and the risk of PJI. The population enrolled in the current study showed 42.86% THAs in Group A and 14.29% THAs in Group B were performed on primary hip osteoarthritis; these data were in contrast with those reported by Ferguson [32], who reported 90% PJIs in THAs for primary, and 10% PJIs in THAs for secondary, osteoarthritis.

No risk factors were found to be associated with Group A or Group B, except for major depression, that was significantly more frequent in Group B (*p* = 0.049). Klement’s et al. [33] observed that patients with mood disorders, or other psychiatric diseases, were prone to develop more medical and surgical complications following a THA. It is easy to understand the connection between PJI and mood disorders, especially depression, considering the psychological burden of disability. PJIs compromise the quality of life of patients, and are a relevant psychological stressor, similar to oncologic diseases. Knebel et al. [34], performed a prospective longitudinal study on 31 patients with PJIs after total knee arthroplasty. Psychometrically-validated standardized questionnaires (like Patient Health Questionnaire (PHQ-4) were used to measure psychosocial stress via self-assessment at four time points: (1) before the explant of the prosthesis; (2) after explant; (3) after the antibiotic treatment and before reimplantation; and (4) three months after reimplantation (follow-up). They found that eighteen out of thirty-one patients (58.1%) showed a PHQ-4 score above the cut-off value for depression at least once during treatment, with the highest score collected before reimplantation. In a retrospective study conducted by Katakam et al. [35], mood disorders, like generalized anxiety disorder or major depressive disorder, were identified as risk factors for failure following debridement and implant retention, performed to treat acute PJI of the hip or knee. Similarly, Cancienne et al. [29] showed that major depression was associated with a higher risk of not having reimplantation after resection arthroplasty. How mood disorders could affect the outcomes of the treatment of PJIs is not completely clear, but it has been demonstrated that stress and depression result in an impairment of the immune response [36]. Moreover, patients affected by major depression tend to show a low adhesion to chronic antibiotic therapy, the mainstay of the treatment of PJIs after resection arthroplasty [37,38].

Patients in Group A showed a profile of PJI sustainers closer to the literature findings, with gram+ germs being responsible for the infection in 71% of patients; this finding was in agreement with the study by Triantafyllopoulos et al. [39], who performed a retrospective study analyzing 36,494 THAs from a single institutional arthroplasty database. They reported that the majority of PJIs on THA were caused by gram+ pathogens. In Group B, the majority of infections were polymicrobial, accounting for 57% of patients. Conversely, in Group A polymicrobial infections represented 14% of the total, in line with previous findings (4–37%) [40]. The association between polymicrobial PJI and worse outcomes has already been reported. Kavolus et al. [41] reported that polymicrobial PJIs are associated with prolonged hospitalization times, increased surgical times, and with a lower success rate of treatment, only reaching 71%. Similar findings were observed by Tan et al. [42], who, in a retrospective observational study, outlined the poorer outcomes of polymicrobial PJIs when compared with monomicrobial or culture negative PJIs. Tan et al. [42] reported that more salvage procedures, like amputations and joint arthrodesis, were observed in a group of polymicrobial PJIs, compared to monomicrobial and culture negative PJIs. The low rate of response to therapy in patients in the Tan study was also associated with an increase in the overall mortality.

## 5. Conclusions

In conclusion, the current study showed that patients with polymicrobial infections and major depression are more prone to worse outcomes after antibiotic therapy, that may hamper THA reimplantation procedures in TSE arthroplasty protocols. Current findings may drive further research and contribute to the understanding of the role of these risk factors in chronic PJI patients.

## Figures and Tables

**Table 1:** Patients’ demographics.

			Group A	Group B
Male to Female Ratio			10:4	4:3
Age at Surgery			51 (31–82)	57.7 (27–86)
Primary Diagnosis for THA				
	Primary Arthritis		6	1
	Secondary			
		AVNH	1	-
		DDH	1	1
	Post Fracture		2	2
	Unknown		4	3
Surgical Approach				
	PL		11	4
	DL		3	2
	DA		-	1
I&D			8/14	5/7

AVNH = avascular necrosis of the hip; DDH = developmental dysplasia of the hip; PL = posterolateral; DL = lateral; DA = anterior.

**Table 2:** Risk factors.

		Group A	Group B
BMI			
	≤25	7	4
	25 < BMI < 30	5	2
	≥30	2	1
ASA score			
	I	1	-
	II	7	4
	III	6	3
	IV	-	-
Diabetes			
	I	1	-
	II	3	1
Alcohol Abuse		-	-
Smoking Habits		5	3
Active infections		3	2
Rheumatic disease		-	-
Malignancy		1 (Breast Cancer)	1 (Chronic Lymphoproliferative syndrome)
IVDA		2	1
Revision Surgery		7	2
CVD	HF AF	3 1	1 -
Mood Disorders		1	3

BMI = body mass index; ASA = American Society of Anesthesiologists; IVDA = intravenous drug abuse; CVD = cardiovascular disease; HF = heart failure; AF = atrial fibrillation.

**Table 3:** Pathogens.

Pathogens	Group A			Group B	
Single					
	Gram+	10	1 *S. Epidermidis*	2	1 *S. Epidermidis*
			3 *S. Aureus*		1 MRSA
			1 MRSE		
			1 MRSA		
			1 *E. Faecalis*		
			1 *S. Pyogenes*		
			1 *S. Capitis*		
			1 *S. Caprae*		
	Gram−	2	1 *E. Cloacae*	1	1 *P. Aerugionsa*
			1 *P. Aeruginosa*		
Polymicrobial		2	1 *C. Striatum* + MRSE	4	1 MRSE + *S. Lugdunensis*
			1 MRSE + *E. Coli* + *S. Haemoliticus*		1 *P. Aeruginosa* + *P. Mirabilis* + *K. Pneumoniae* + *S. Aureus*
					1 *S. Aureus* + *P. Aeruginosa*
					1 *S. Aureus* +Drug resistant gram−

MRSE = methicillin resistant Staphylococcus epidermidis; MRSA = methicillin-resistant Staphylococcus aureus.

## Data Availability

All collected data are reported in the current manuscript.
